# Effect of loss of control effectiveness on an inverted pendulum balanced on a moving quadrotor

**DOI:** 10.1016/j.heliyon.2023.e14494

**Published:** 2023-03-15

**Authors:** Jackson Oloo

**Affiliations:** Department of Electrical and Electronic Engineering, Jomo Kenyatta University of Agriculture and Technology, P.O Box 62000-00200, Nairobi Kenya

**Keywords:** Rotor failure, Adaptive MPC, LQR controller, Weighting matrices, Loss of effectiveness

## Abstract

This paper investigates the effect of loss of rotor effectiveness on the states of an inverted pendulum mounted at the center of mass of a moving quadrotor. An adaptive Model Predictive Controller is utilized to develop a controller that enables the quadrotor to track a circular trajectory while experiencing varied degrees of loss of actuator effectiveness. Nominal states of the quad-pendulum system on a circular trajectory are determined from the investigated dynamic equilibria. The performance of the developed fault tolerant controller against the pendulum states is compared with LQR performance in numerical simulations. Recommendations to improve performance against the observed errors are highlighted.

## 1 Introduction

As a classical control problem, the inverted pendulum presents a highly nonlinear system. It has been extensively used to demonstrate advances in optimization techniques such as neural networks [1], fuzzy logic [2] and reinforcement learning [3]. Quadrotor trajectory tracking and arbitrary orientation has been extensively studied [4,5]. Quadcopter-pendulum system is thus a highly demonstrative exercise that presents agile capabilities of quadcopters [6]. In modern set ups, quadcopters have been commercially utilized to transport loads over considerable distances. The loads attached or placed at the center of the quadrotor, can always be considered as under actuated systems. Therefore, the inverted pendulum can be modelled as the simplest payload attached on a quadcopter. Therefore, it is imperative to address the stability of such a system with a potential loss of control effectiveness across all the quadcopter rotors. There has been extensive research around suspended payloads using multi-UAVs in recent times, [[7], [8], [9]]. However, such suspended payloads are likely to be damaged in the landing process of the quadcopters. It is therefore important that the dynamics of the mounted payloads on top of quadrotor systems be studied to realize feasible control actions especially for any potential quadrotor actuator faults.

This paper is motivated by the work accomplished in Refs. [6,10] to bring an inverted pendulum to equilibrium but under potential actuator faults. Feedback regularization is used in Ref. [11] with a simple PD system for stabilization. The control strategy presented acceptable performances in stabilizing the quadrotor with a yaw orientation that ensures upright position of the inverted pendulum. However, one of the author’s recommendations is the need for careful examination of the effect of disturbances on stability of their system.

The contributions of this paper are: 1. Modelling the quad-pendulum system under quadrotor actuator loss of effectiveness with the objective of stabilizing the system under dynamic equilibria. In Ref. [6], an independent subsystem is considered by utilizing inputs from rotational angular rates. In this article, a large, coupled multi-input multi-output system is considered through rotational inertia and input thrust from each rotor. 2. Design of a control action for a linearized quad-pendulum system under quadcopter rotor failure. Weighting matrices for the terminal and stage costs are carefully chosen through iterative tuning process for the MPC problem being solved. The controller is based on adaptive model predictive controller where a general cost function is minimized and the value of prediction horizon N is updated appropriately for every iteration.

This paper is presented with the following sections: Section I introduces the topic of study and the available literature in this topic, Section II describes the dynamic model of the quad-pendulum system, the controller is designed in Section III and numerical simulation using MATLAB is performed in Section IV. Here, the performance of the designed controller is validated using LQR controller under different magnitudes of quadcopter rotor fault. Section V highlights the conclusion with observations and future directions.

## 2 Dynamic modelling

### 2.1 Modelling of quadrotor actuator faults

The state space representation of a quadrotor model can be written as [12]

(1) $$\overset{•}{\begin{array}{l} x=Ax+Bu\\ y=Cx \end{array}}$$

With $x\in R^{n}$, $u\in R^{m}$ are the state vector and control input respectively. $y\in R^{p}$ is the system output.

The derivation of the model for a quadrotor under actuator fault is based on [12], where the faulty model is represented as:

(2) $$\overset{•}{\begin{array}{l} x=Ax+B_{f}u\\ y=Cx \end{array}}$$

and $B_{f}=BL_{f}$ as the after-fault control input where $L_{f}=diag\left\{l_{f1}...l_{fm}\right\}$. $l_{f}$ indicates loss of rotor effectiveness where for $l_{fi}=1$, the actuator is fault free and $l_{fi}=0$, represents complete failure of the actuator. The effectiveness factor can thus be varied as $0\leq l_{f1}\leq 1$.

### 2.2 Inverted pendulum

Assumption 1 The resultant forces of the inverted pendulum on the quadcopter are negligible since its mass is assumed to be small relative to that of the quadcopter.

Assumption 2 The quadcopter dynamics are independent of the pendulum whereas the motion of the quadcopter influences the dynamics of the pendulum. However, the pendulum is modelled as inertia less point mass where base motion is not caused by the quadrotor rotations [6].

The two degrees of freedom for the pendulum are described by position of its center of mass with respect to the base of attachment in the inertial coordinate system. The Langrangian equation for the pendulum’s kinetic and potential energy is given as in equation (3), [6]. The equation is used to describe the states of the pendulum’s dynamic system relative to its positional coordinates.

(3) $$\Theta =\frac{1}{2}\left({\left(\overset{•}{x}+\overset{•}{r}\right)}^{2}+{\left(\overset{•}{y}+\overset{•}{s}\right)}^{2}+{\left(\overset{•}{z}-\frac{r\overset{•}{r}+s\overset{•}{s}}{\psi }\right)}^{2}\right)-g\left(z+\psi \right)$$

where r and s are the translational position of the center of mass of the pendulum with respect to its base along the x-axis and y-axis respectively. $\psi :\sqrt{L^{2}-r^{2}-s^{2}}$ is the pendulum’s relative position along the z axis. rands are the translational position along the x-axis and y-axis respectively.

L denotes the length from the center of mass to the pendulum base. Equation (3) can be utilized to describe the equations of motion of the inverted pendulum as follows:

(4) ddt(∂Θ∂r•)−∂Θ∂r=0

(5) ddt(∂Θ∂s•)−∂Θ∂s=0

### 2.3 Quad-pendulum dynamics

The combined dynamics are based on derivations from Ref. [6], where the controlled translational acceleration of the quadrotor is responsible for linear motion of the quadrotor and the pendulum’s motion through nonlinear dynamic equations.

(6) [x••y••z••]=RVVO(α,β,γ)[00τ]+[00−g]

where τ is the collective thrust.

(7) $$\left[\begin{array}{c} \overset{•}{\gamma }\\ \overset{•}{\beta }\\ \overset{•}{\alpha } \end{array}\right]={\left[\begin{array}{ccc} \cos\left(\beta \right)\cos\left(\gamma \right) & -\sin\left(\gamma \right) & o\\ \cos\left(\beta \right)\sin\left(\gamma \right) & \cos\left(\gamma \right) & 0\\ -\sin\left(\beta \right) & 0 & 1 \end{array}\right]}^{-1}\left[\begin{array}{c} \omega_{x}\\ \omega_{y}\\ \omega_{y} \end{array}\right]$$

Equations (4), (5) results into

(8) $$\left[\begin{array}{c} \overset{••}{r}\\ \overset{••}{s} \end{array}\right]=k\left(r,s,\overset{•}{r},\overset{•}{s},\overset{••}{x},\overset{••}{y},\overset{••}{z}\right)$$

The function k is derived in detail in Ref. [6].

The motor thrusts for relative upward velocity and rotational velocity relates as follows:

(9) $$\left[\begin{array}{c} \omega_{x}\\ \omega_{y}\\ \omega_{z}\\ \overset{•}{v_{z}} \end{array}\right]=\left[\begin{array}{cccc} -I_{xx}^{-1} & I_{xx}^{-1} & 0 & 0\\ 0 & 0 & -I_{xx}^{-1} & I_{xx}^{-1}\\ -I_{zz}^{-1} & -I_{zz}^{-1} & -I_{zz}^{-1} & I_{zz}^{-1}\\ m^{-1} & m^{-1} & m^{-1} & m^{-1} \end{array}\right]\left[\begin{array}{c} F_{1}\\ F_{2}\\ F_{3}\\ F_{4} \end{array}\right]$$

where α,β,γ are the yaw, pitch and roll angles

Circular trajectory tracking under actuator loss of effectiveness is investigated in this paper with the control input is described as:

(10) $$u=\left[\begin{array}{c} T_{1}\\ T_{2}\\ T_{3}\\ T_{4} \end{array}\right]$$

For $B_{f}=BL_{f}$ the control effectiveness is varied between $0\leq l_{f1}\leq 1$. Therefore, the input varied according to equation (2). “Initial Equation (11) *has been deleted as suggested by Reviewer #1: Comment 3, the subsequent equations have thus changed in numbering”*

However, for a circular trajectory of radius R, the system can be linearized around this point as described in [13]

$$\overset{••}{\tilde{p}}=\frac{\psi_{0}^{2}}{L^{2}}\left[\tilde{p}\left(\Phi^{2}+\frac{gL^{2}}{\psi_{0}^{3}}\right)+2\overset{•}{\tilde{q}}\Phi +\tilde{\mu }\left(-\frac{p_{0}}{\psi_{0}}a_{0}\;\sin\;\mu_{0}-a_{0}\;\cos\;\mu_{0}\right)+\tilde{a}\left(\frac{p_{0}}{\psi_{0}}a_{0}\;\cos\;\mu_{0}-\sin\;\mu_{0}\right)\right]$$

(11) $$\overset{••}{\tilde{q}}=\tilde{q}\left(\Phi^{2}+\frac{g}{\zeta_{0}}\right)-2\tilde{p}\Phi +\tilde{v}a_{0}$$

$$\overset{••}{\tilde{u}}=\tilde{a}\sin\;\mu_{0}+\tilde{\mu }a_{0}\;\cos\;\mu_{0}+2\overset{•}{\tilde{\upsilon }\Phi +\tilde{u}\Phi^{2}}$$

$$\overset{••}{\tilde{\upsilon }}=-\tilde{v}a_{0}-2\overset{•}{\tilde{u}\Phi +\tilde{\upsilon }\Phi^{2}}$$

$$\overset{••}{\tilde{w}}=\tilde{a}\cos\;\mu_{0}-\tilde{\mu }a_{0}\;\sin\;\mu_{0}$$

where μ,ν and a are the Euler angles and the compounded thrust.

u, υ and w describes quadcopter’s equation of motion as

$$\left[\begin{array}{c} u\\ \upsilon \\ w \end{array}\right]={\left[\begin{array}{ccc} \cos\left(\Phi t\right) & -\sin\left(\Phi t\right) & 0\\ \sin\left(\Phi t\right) & \cos\left(\Phi t\right) & 0\\ 0 & 0 & 1 \end{array}\right]}^{-1}\left[\begin{array}{c} x\\ y\\ z \end{array}\right]$$

with Φ as the rotational rate around a given circular trajectory which the quadrotor is supposed to track. p and q can be derived from $\left[\begin{array}{c} r\\ s \end{array}\right]=\left[\begin{array}{cc} \cos\;\Omega t & -\sin\;\Omega t\\ \sin\;\Omega t & \cos\;\Omega t \end{array}\right]\left[\begin{array}{c} p\\ q \end{array}\right]$ with Ω the constant rotational rate.

## 3 Controller design

Without loss of generality, Equation (12) can be expressed as linear state space equation as follows:

(12) $$\overset{•}{e}=A\left(R\left(t\right),\Phi \left(t\right)\right)e+B\left(R\left(t\right),\Phi \left(t\right)\right)u$$

where e is the deviation from the defined trajectory.

In [14], the cost function for solving the MPC problem has been defined as:

(13) $$J\left(x_{0},u\right)=\sum_{K=0}^{N-1}l\left(x\left(k\right),u\left(k\right)\right)+V_{f}\left(x\left(N\right)\right)$$

where u∈U,x∈X are the sets of input and state constraints respectively.

l(x,u) and $V_{f\left(x\right)}$ are the stage cost and terminal penalty respectively, these can be expressed as:

(14) $$\begin{array}{l} l\left(x\left(k\right),u\left(k\right)\right)=x{\left(k\right)}^{T}Qx\left(k\right)+u{\left(k\right)}^{T}Ru\left(k\right)\\ V_{f}\left(x\left(N\right)\right)=x{\left(N\right)}^{T}Px\left(N\right) \end{array}$$

From Equation (13), an adaptive MPC controller is utilized to account for R, the radius of the circular trajectory and Ω, the rotational velocities. Equation (14) is used to solve the MPC problem but the prediction matrices are updated for every iteration with respect to current reference velocity and circular trajectory radius as detailed in Ref. [13].

The equilibrium point is defined as a hovering point where the pendulum balances vertically at the center of mass of the quadrotor. Equation (14) represents the optimization problem where the stage cost and the penalty cost are expressed in Equation (15). P is determined by discrete algebraic process covered in Ref. [13]. Q and R are weighting matrices that are determined through iterative tuning process.

## 4 Numerical simulation results

### 4.1 Simulation with 40% actuator loss of effectiveness

The numerical simulations in this section were performed using MATLAB. The quad-pendulum system parameters used in this paper are similar to those validated experimentally in Ref. [6]. The simulation was conducted for different values of N. N was varied between 7 and 25. As N is decreased up to a certain point, settling time and undershoot decreases, however, the pendulum is offset away from the 0-equilibrium point. A higher value of N brings the pendulum closer to the 0 equilibrium point but with increased settling times.

**Fig. 1:**
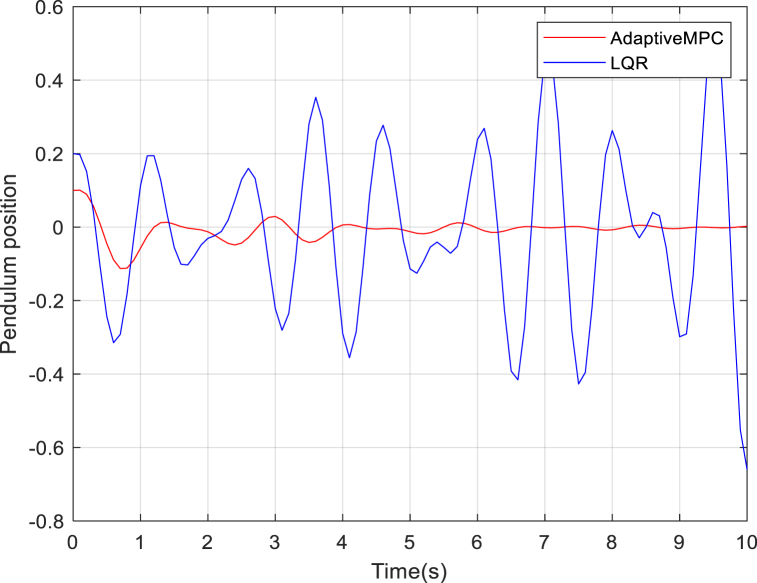
Pendulum behavior around the 0-equilibrium point for 40% quadcopter actuator loss of effectiveness. The pendulum could not be balanced with LQR controller at 40% fault. The pendulum position error can be seen to increase with time.

For different values of N and the iteratively determined values of Q and R, the quad-pendulum system could not be stabilized for values higher than 50% of loss of actuator effectiveness for both the adaptive model predictive controller and the LQR controller. However, from Fig. 1, at 40% loss of actuator effectiveness across all rotors, the adaptive controller was able to bring the pendulum around the 0-equilibrium point although sustaining steady state errors with a longer settling time. The LQR controller was unable to stabilize the pendulum, which continually oscillated from the equilibrium point. The oscillations further from the reference point could be due to increase of quadrotor speed around the circular trajectory.

**Fig. 2:**
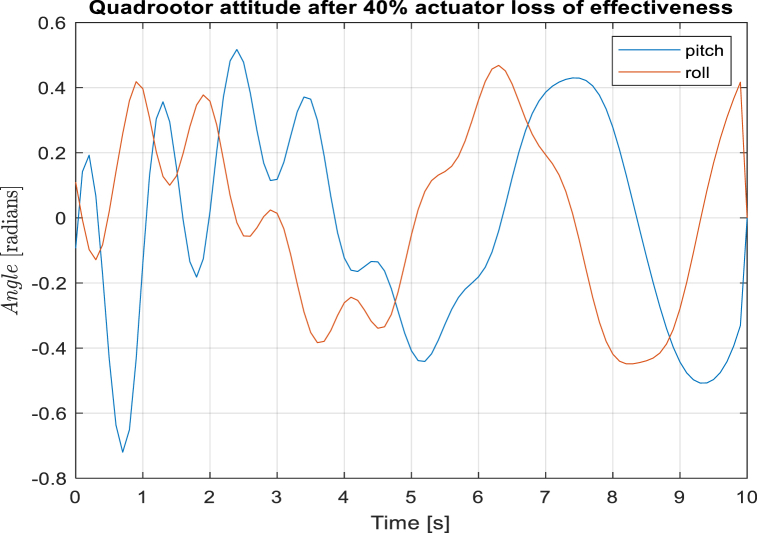
Quadrotor attitude control with adaptive MPC. The pitch and roll angles have a limit of 0.5 radians.

**Fig. 3:**
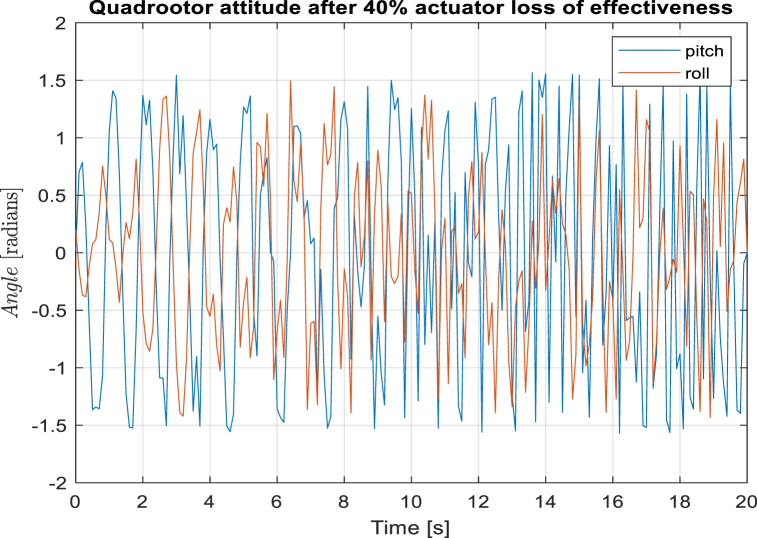
Quadrotor attitude control with LQR controller.

Fig. 2 shows that the quadrotor regains stability after sometime since the weighting matrices for the stage cost are iteratively tuned for the structural changes. The quadrotor, under LQR controller, is however uncontrollable for 40% actuator loss as shown in Fig. 3.

### 4.2 Simulation with 30% actuator loss of effectiveness

**Fig. 4:**
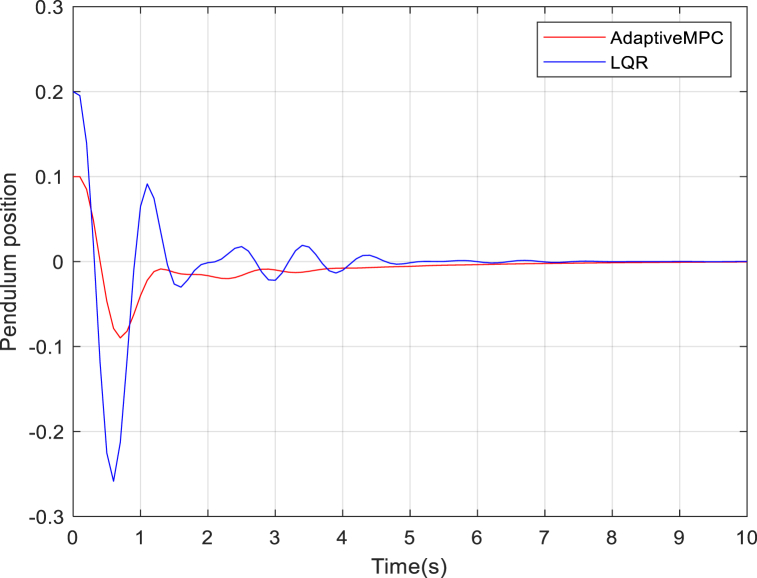
Pendulum behavior around the 0-equilibrium point for 30% quadcopter actuator loss of effectiveness.

From Fig. 4, the LQR controller was able to stabilize the quad-pendulum system although with a longer settling time, 6s, and larger overshoots as compared to the adaptive MPC, 4s.

**Fig. 5:**
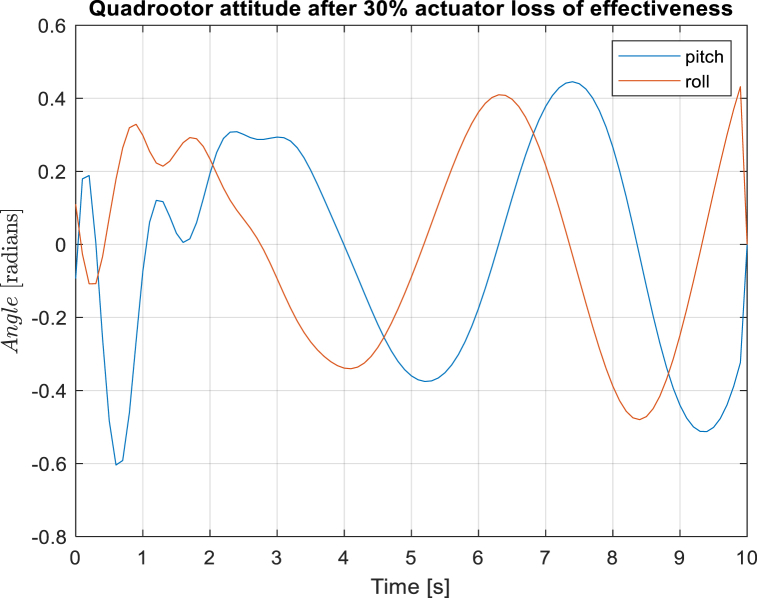
Quadcopter pitch and roll angles at 30% actuator loss of effectiveness with adaptive model predictive controller.

**Fig. 6:**
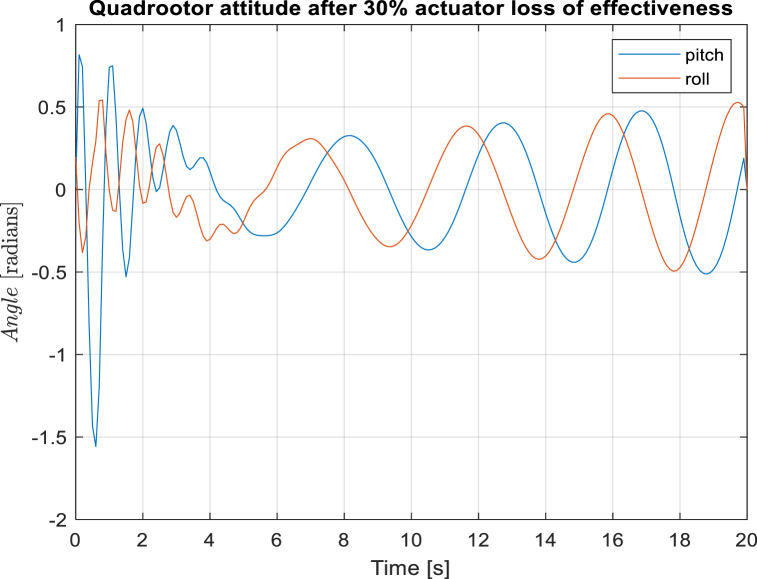
Quadcopter pitch and roll angles at 30% actuator loss of effectiveness with LQR controller.

Fig. 5, Fig. 6 depicts the quadcopter attitude control. The LQR controller, due to initial conditions, sustains larger overshoots beyond the 0.5 radians limit with a settling time of 5s while the designed controller sustains lower settling time of 3s and constraints the attitude within the 0.5 limit even with the quadrotor structural disturbances.

## 5 Conclusion

The designed controller was able to stabilize the quad-pendulum system around the equilibrium point as compared to the LQR controller at 40% actuator loss of effectiveness. It was observed that manually increasing the value of N drives the system to stability up to a certain point and then it starts becoming unstable again. However, this process seems tedious and time consuming. For the sustained systematic disturbance, an approximator could be applied to automatically process the value of N online.

## Author contribution statement

Jackson Ochieng Oloo: Conceived and designed the experiments; Performed the experiments; Analyzed and interpreted the data; Contributed reagents, materials, analysis tools or data; Wrote the paper.

## Funding statement

This research did not receive any specific grant from funding agencies in the public, commercial, or not-for-profit sectors.

## Data availability statement

No data was used for the research described in the article.

## Declaration of interest’s statement

The authors declare no conflict of interest.
